# Cerebrospinal Fluid Penetration of Ceftolozane-Tazobactam in Critically Ill Patients with an Indwelling External Ventricular Drain

**DOI:** 10.1128/AAC.01698-20

**Published:** 2020-12-16

**Authors:** Fekade B. Sime, Melissa Lassig-Smith, Therese Starr, Janine Stuart, Saurabh Pandey, Suzanne L. Parker, Steven C. Wallis, Jeffrey Lipman, Jason A. Roberts

**Affiliations:** a University of Queensland Centre for Clinical Research, Faculty of Medicine, The University of Queensland, Brisbane, Australia; b School of Pharmacy, Centre for Translational Anti-Infective Pharmacodynamics, The University of Queensland, Brisbane, Australia; c Department of Intensive Care Medicine, Royal Brisbane and Women’s Hospital, Brisbane, Australia; d Division of Anaesthesiology Critical Care Emergency and Pain Medicine, Nîmes University Hospital, University of Montpellier, Nîmes, France; e Pharmacy Department, Royal Brisbane and Women’s Hospital, Brisbane, Australia

**Keywords:** ceftolozane-tazobactam, cerebrospinal fluid, CSF penetration, meningitis, ventriculitis, ceftolozane, tazobactam

## Abstract

The aim of this study was to describe the pharmacokinetics of ceftolozane-tazobactam in plasma and cerebrospinal fluid (CSF) of infected critically ill patients. In a prospective observational study, critically ill patients (≥18 years) with an indwelling external ventricular drain received a single intravenous dose of 3.0 g ceftolozane-tazobactam. Serial plasma and CSF samples were collected for measurement of unbound ceftolozane and tazobactam concentration by liquid chromatography. Unbound concentration-time data were modeled in R using Pmetrics. Dosing simulations were performed using the final model. A three-compartment model adequately described the data from 10 patients. For ceftolozane, the median (interquartile range [IQR]) area under the unbound concentration-time curve from time zero to infinity (*f*AUC_0-inf_) in the CSF and plasma were 30 (19 to 128) h·mg/liter and 323 (183 to 414) h·mg/liter, respectively. For tazobactam, these values were 5.6 (2 to 24) h·mg/liter and 52 (36 to 80) h·mg/liter, respectively. Mean ± standard deviation (SD) CSF penetration ratios were 0.2 ± 0.2 and 0.2 ± 0.26 for ceftolozane and tazobactam, respectively. With the regimen of 3.0 g every 8 h, a probability of target attainment (PTA) of ≥0.9 for 40% *f*T_>MIC_ in the CSF was possible only when MICs were ≤0.25 mg/liter. The CSF cumulative fractional response for Pseudomonas aeruginosa-susceptible MIC distribution was 73%. The tazobactam PTA for the minimal suggested exposure of 20% *f*T_>1 mg/liter_ was 12%. The current maximal dose of ceftolozane-tazobactam (3.0 g every 8 h) does not provide adequate CSF exposure for treatment of Gram-negative meningitis or ventriculitis unless the MIC for the causative pathogen is very low (≤0.25 mg/liter).

## Abstract (precis)

The aim of this study was to describe the pharmacokinetics of ceftolozane-tazobactam in plasma and cerebrospinal fluid (CSF) of infected critically ill patients. In a prospective observational study, critically ill patients (≥18 years) with an indwelling external ventricular drain received a single intravenous dose of 3.0 g ceftolozane-tazobactam. Serial plasma and CSF samples were collected for measurement of unbound ceftolozane and tazobactam concentration by liquid chromatography.

## INTRODUCTION

Gram-negative nosocomial meningitis and ventriculitis are likely to occur in critical care settings, often associated with brain trauma, brain surgery, or insertion of spinal fluid shunts after brain surgery or in patients with severe urosepsis (1). Gram-negative meningitis is particularly challenging for treatment when some of the common etiologic bacteria encountered (e.g., Acinetobacter baumannii, Pseudomonas aeruginosa, and Enterobacter aerogenes) exhibit relatively high MICs within the susceptibility range (2). The initial empirical therapy recommendation for patients with healthcare-associated ventriculitis and meningitis is an antipseudomonal beta-lactam antibiotic in combination with vancomycin (3).

Ceftolozane-tazobactam is a relatively novel antipseudomonal beta-lactam antibiotic with activity against Gram-negative bacteria, including multidrug-resistant P. aeruginosa (4, 5). It is approved for the treatment of complicated urinary tract infections, complicated intra-abdominal infections, hospital-acquired bacterial pneumonia, and ventilator-associated pneumonia (6). Off label, its clinical use extends to other clinical conditions, including as a rescue treatment for central nervous system infections (CNS) caused by extensively drug-resistant (XDR) P. aeruginosa unresponsive to other potent beta-lactams, such as meropenem (7–9). However, the effective use of ceftolozane-tazobactam for these infections is uncertain due to a lack of data on cerebrospinal fluid (CSF) penetration. Currently, no dosing guidelines exist for use in CNS infections.

The aim of this study was to describe the plasma and CSF population pharmacokinetics of ceftolozane and tazobactam and evaluate the adequacy of CSF exposure from intravenous dosing regimens (3 g every 8 h [q8h], 3-g loading dose plus 9-g continuous infusion) in the treatment of critically ill patients with an indwelling external ventricular drain (EVD).

## RESULTS

The study enrolled 10 patients; Table 1 summarizes their demographic and clinical characteristics. Five patients had confirmed/suspected CNS infection. Paired plasma versus CSF observed concentration-time profiles for each patient are given in Fig. S1 and S2 in the supplemental material for ceftolozane and tazobactam, respectively.

**TABLE 1:** Demographic and clinical characteristics of study participants^*a*^

Patient no.	Sex	Age (yr)	wt (kg)	Serum creatinine (μmol/liter)	Urinary creatinine clearance (ml/min/1.73m^2^)	Albumin (g/liter)	ALT (IU/ml)	AST (IU/ml)	ALP (IU/ml)	APACHE II	SOFA	Site of infection	Organism
1	Female	62	75	36	199	25	41	36	76	27	5	Lung	Haemophilus influenzae
2	Female	52	50	43	98	18	26	38	206	21	3	CNS	Escherichia coli
3	Female	59	65	47	102	29	42	27	98	13	0	UTI	Proteus mirabilis
4	Female	25	55	34	192	19	21	31	58	25	2	CNS	Staphylococcus epidermidis
5	Female	84	65	66	104	25	19	14	57	17	3	Lung	Streptococcus pneumoniae
6	Male	64	78	140	37	22	83	59	112	13	3	CNS*	Unknown
7	Male	58	90	50	155	23	46	17	84	12	3	Lung	Staphylococcus aureus, Haemophilus influenzae, Streptococcus pneumoniae
8	Male	27	95	59	190	26	9	2.5	62	17	5	CNS*	Unknown
9	Female	65	75	54	101	21	77	37	84	18	3	CNS*	Unknown
10	Female	78	90	42	113	28	22	16	51	23	5	Unknown	Unknown
Median		60.5	75	48.5	108.5	24	33.5	29	80	17.5	3		
Q1		53.5	65	42.25	101.25	21.25	21.25	16.25	59	14	3		
Q3		64.75	87	57.75	181.25	25.75	45	36.75	94.5	22.5	4.5		

a ALT, alanine aminotransferase; AST, aspartate aminotransferase; ALP, alkaline phosphatase; APACHE II, Acute Physiology and Chronic Health Evaluation II score; SOFA, sequential organ failure assessment score; CNS, central nervous system; UTI, urinary tract infection; *, suspected; Q1, first quartile; Q3, third quartile.

**FIG 1:**
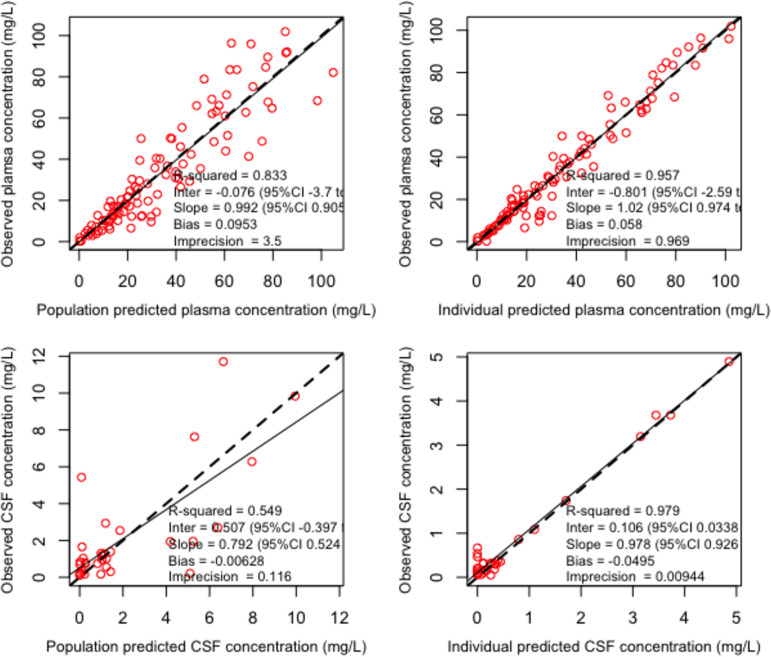
Observed versus predicted concentration diagnostic plot for ceftolozane. The solid lines represent the line of best fit (regression line). The dashed lines represent the line of identity. CI, confidence interval.

**FIG 2:**
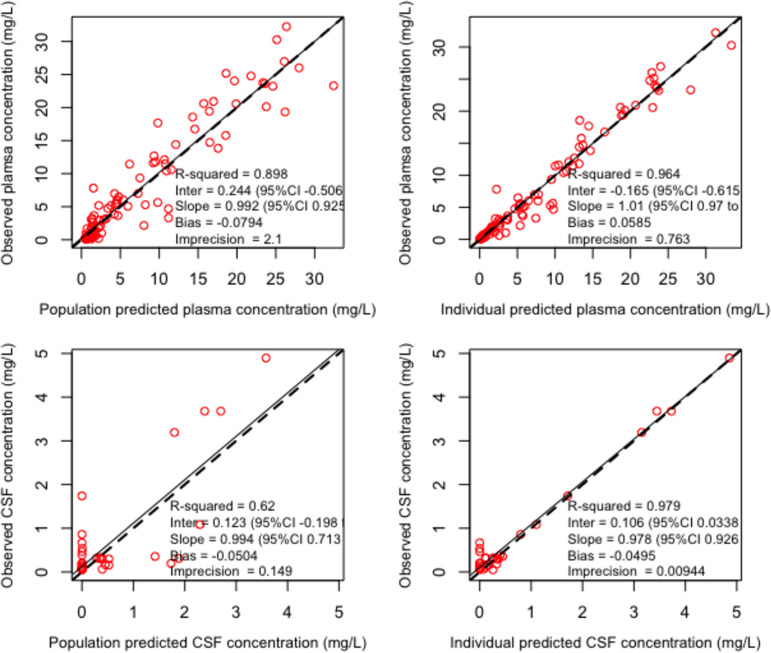
Observed versus predicted concentration diagnostic plot for tazobactam. The solid lines represent the line of best fit (regression line). The dashed lines represent the line of identity. CI, confidence interval.

**FIG 3:**
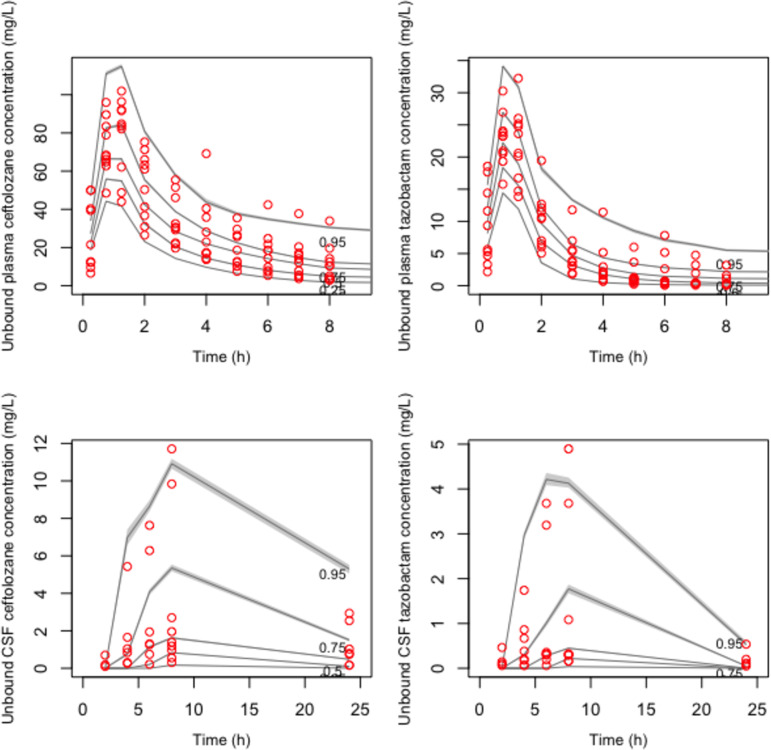
Visual predictive check plots. The small circles indicate observed concentrations. The smooth lines represent simulated concentrations at the designated quantile, given by the number on top of the line. The shaded zone on each line represents the 95% confidence interval.

The schematic representation of the final structural model is given in the supplemental material (Fig. S3). Creatinine clearance on plasma clearance (CL_plasma_) and body weight on volumes of distribution of the central compartment (*V_c_*) and the CSF compartment (*V*_CSF_) were the only covariates that improved model fit (better diagnostic plots and reduced objective function values) for both ceftolozane and tazobactam. CL_plasma_ was linearly related to creatinine clearance normalized to the study population average as CL_plasma_ = CL × (CL_CRurinary_/129), where CL is the typical value of clearance and CL_CRurinary_ is measured urinary creatinine clearance. Similarly, for both drugs, *V_c_* and *V*_CSF_ were linearly related to body weight (wt) normalized by the study population average as *V_c_* = *V_c_* × (wt/74) and *V*_CSFcompartment_ = *V*_CSF_ × (wt/74), where *V_c_* and *V*_CSF_ are typical values. Residual error or uncertainty associated with the assay (standard deviation [SD] of observations/concentrations) was best described by first-degree polynomial functions: SD = 0.5 × [Obs] + 0.01 and error = 0.5 × [Obs] + 0.1 for plasma and CSF observations (concentrations) [Obs]. Residual unexplained source of variability (error) was described by error = (SD^2^ + 0.8)^0.5^. Parameter estimates for the final models are given in Table 2. Estimated mean systemic CL was 7.2 and 16.6 liters/h for ceftolozane and tazobactam, respectively. The average lag time for penetration of ceftolozane and tazobactam was 4.3 and 4.8 h, respectively. Only about 25% and 18% of the intravenous ceftolozane and tazobactam dose, respectively, penetrated into the CSF. Percent shrinkage was reasonably low for all parameters, suggesting that the data were adequate to estimate the individual parameters. The parameter distribution plots are given in Fig. S4 and S5. The observed versus predicted diagnostic plots for the final models are given in Fig. 1 and 2 for ceftolozane and tazobactam, respectively. The visual predictive check plots of the final covariate models (*n* = 1,000) are given in Fig. 3. Except for a few outliers, nearly all observations fall between the 5th and 95th percentiles of simulations, suggesting adequate predictive performance of the model.

**TABLE 2:** Parameter estimates for the final ceftolozane and tazobactam models^*a*^

Parameter (unit)	Value for:							
	Ceftolozane				Tazobactam			
	Mean	SD	Median	Shrink (%)	Mean	SD	Median	Shrink (%)
CL (liters/h)	7.205	1.504	7.372	2.968	16.563	6.539	14.771	2.914
*V_c_* (liter)	16.154	3.616	17.609	0.14	23.001	6.647	22.162	0.915
*K*_cp_ (h^−1^)	0.474	0.366	0.461	0.745	0.521	0.323	0.506	1.953
*K*_pc_ (h^−1^)	0.535	0.316	0.724	0.413	0.308	0.38	0.076	0.814
*K*_e_csf_ (h^−1^)	0.23	0.173	0.139	0.365	0.694	0.283	0.662	7.434
*V*_CSF_ (liter)	142.015	34.26	161.97	0.869	127.308	38.851	114.213	6.057
*T*_lag_csf_ (h)	4.348	1.923	3.931	0.989	4.801	1.973	5.652	0.825
*K*_p_csf_ (h^−1^)	0.588	0.74	0.342	0.08	0.846	0.761	0.467	16.119
*F*_csf_	0.253	0.18	0.188	0.283	0.18	0.068	0.19	10.783

a SD, standard deviation; CL, clearance; *V_c_*, apparent central volume of distribution; *K*_cp_, rate constant for the transfer from the central compartment to the peripheral compartment; *K*_pc_, rate constant for the transfer from the peripheral compartment to the central compartment; *K*_e_csf_, rate constant for elimination from the CSF compartment; *T*_lag_csf_, lag time for penetration into the CSF compartment; *K*_p_csf_, rate constant for penetration into the CSF compartment; *F*_csf_, bioavailability in the CSF compartment.

**FIG 4:**
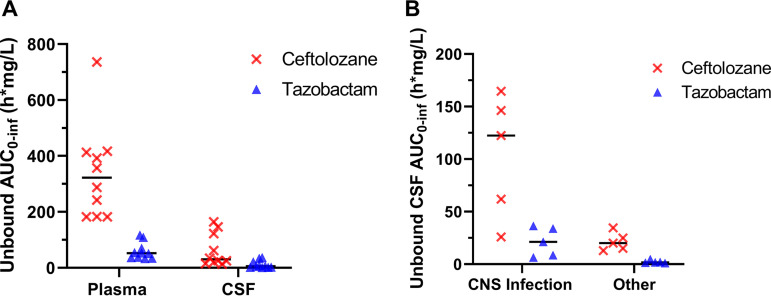
Comparison of plasma versus CSF exposures by AUC_0–inf_ (A) and comparison of CSF exposure between patients with CNS infection versus those without CNS infection by AUC_0–inf_ (B). AUC_0–inf_ was estimated from individual posterior model predictions by the linear trapezoidal method. Horizontal bars indicated median values.

**FIG 5:**
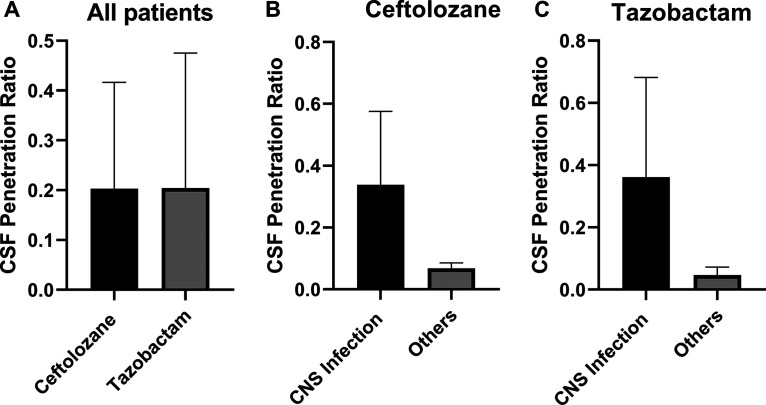
Cerebrospinal fluid (CSF) penetration ratio of ceftolozane and tazobactam (A) and comparison of CSF penetration between patients with CNS infection (*n* = 5) and without CNS infection (*n* = 5) for ceftolozane (B) and tazobactam (C).

Relative to plasma, the overall exposure in the CSF was low for both ceftolozane and tazobactam (Fig. 4A). For ceftolozane, the median (interquartile range [IQR]) area under the unbound (free) concentration-time curve from time zero to infinity (*f*AUC_0–inf_) in the CSF was 30 (19 to 128) h·mg/liter, more than 10-fold lower than the corresponding plasma value of 323 (183 to 414) h·mg/liter. For tazobactam, the median (IQR) *f*AUC_0–inf_ in the CSF was 5.6 (2 to 24) h·mg/liter compared to a ∼10-fold higher value in plasma, 52 (36 to 80) h·mg/liter. The CSF exposure for those patients with confirmed/suspected CNS infection compared to those with other sites of infection appeared higher for both ceftolozane and tazobactam (Fig. 4B). Unpaired *t* test with Welch’s correction for this difference in CSF *f*AUC_0–inf_ was significant for both ceftolozane and tazobactam (*P = *0.0332 and 0.0368, respectively). The CSF penetration ratios estimated from the CSF-to-plasma *f*AUC_0–inf_ ratio were highly variable but comparable for ceftolozane and tazobactam, with a mean ± SD of 0.2 ± 0.2 and 0.2 ± 0.26, respectively (Fig. 5A). Similar to the *f*AUC_0–inf_, CSF penetration ratios were relatively higher for patients with CNS infection (Fig. 5B and C); however, Welch’s *t* test showed no statistically significant difference at an α value of 0.05 (*P *values of 0.0640 and 0.0928 for ceftolozane and tazobactam, respectively).

**FIG 6:**
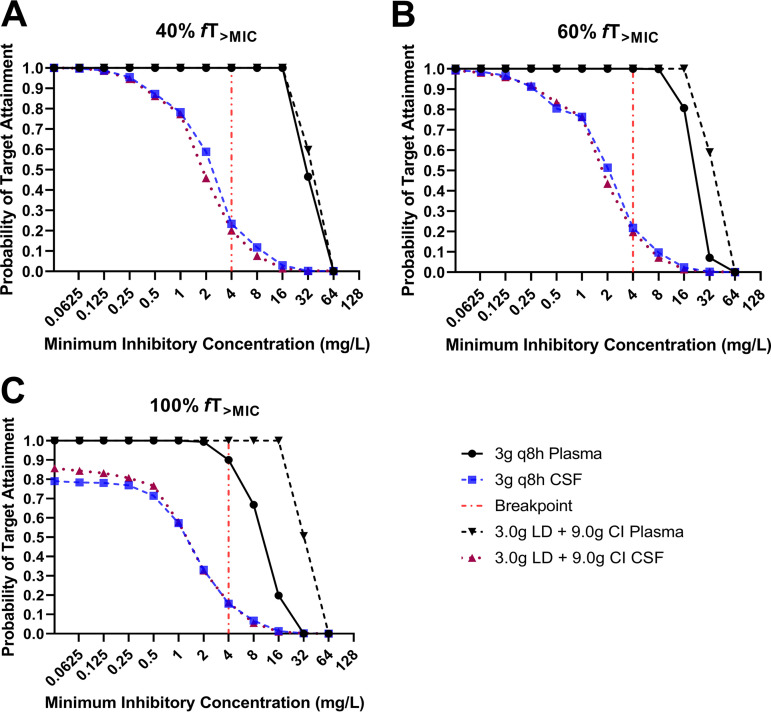
Probability of pharmacokinetic/pharmacodynamic target attainment for ceftolozane in plasma and cerebrospinal fluid (CSF) at pharmacokinetic steady state (120 to 144 h). %*f*T_>MIC_, the percentage of time free ceftolozane concentration is above the MIC; q8h, every 8 h; LD, loading dose; CI, continuous infusion. (A) 40% *f*T_>MIC_. (B) 60% *f*T_>MIC_. (C) 100% *f*T_>MIC_.

The low average penetration ratio of 0.2 resulted in low probability of attaining the recommended % *f*T_>MIC_ targets for ceftolozane in the CSF, unlike the high probability for plasma exposure (Fig. 6). With the 3.0 g q8h (every 8 h) intermittent ceftolozane-tazobactam (2:1 ratio) regimen, dosing simulations showed that an acceptable probability of target attainment (PTA) of ≥0.9 for the 40% and 60% *f*T_>MIC_ targets was attained in the CSF only when MICs were ≤0.25 mg/liter. The more conservative target of 100% *f*T_>MIC_ was not attained even for the lowest MIC in the EUCAST distribution (0.032 mg/liter). A 24-h continuous infusion of 9.0 g with an initial 3.0-g loading did not improve the % *f*T_>MIC_ in the CSF, resulting in a similar rate of target attainment with the intermittent regimen. Similar to ceftolozane, the probability of attaining previously proposed exposure targets for tazobactam was also extremely low in the CSF (see Table 4). The cumulative fractional response (CFR) for P. aeruginosa susceptible MIC distribution (Table 3) consistently reflects suboptimal exposure in the CSF with the high-dose regimens of 3.0 g q8h currently approved for use in humans. A further 50% increase in dose to 4.5 g q8h did not result in ≥85% CFR.

**TABLE 3:** Cumulative fractional response against P. aeruginosa MIC distribution within the susceptibility range for steady-state ceftolozane exposure^*a*^

Ceftolozane-tazobactam dose (2:1 ratio)	% CFR by PK/PD target for:					
	Plasma exposure			CSF exposure		
	40% *f*T_>MIC_	60% *f*T_>MIC_	100% *f*T_>MIC_	40% *f*T_>MIC_	60% *f*T_>MIC_	100% *f*T_>MIC_
3.0 g q8h	100	100	99	73	68	55
3.0 g LD + 9.0 g CI	100	100	100	70	68	57
4.5 g q8h	100	100	100	81	76	65
4.5 g LD + 13.5 g CI	100	100	100	78	76	69

a PK, pharmacokinetic; PD, pharmacodynamic; CFR, cumulative fractional response; q8h, every 8 h, intermittent infusion (1 h); % *f*T_>MIC_, percentage of time free drug concentration is above the MIC; LD, loading dose over 1 h; CI, continuous infusion over 24 h.

## DISCUSSION

In this study, we have shown that, like various other beta-lactams, intravenous ceftolozane and tazobactam inadequately penetrate the CSF. This observation is not unexpected, given the physicochemical properties of both of these drug molecules (hydrophilicity) and that lipid solubility is a major determinant of antibiotic penetration across the blood-brain barrier (BBB) via passive diffusion (10, 11).

It is worth noting that in the present study, half of the participants had presumed or confirmed ventriculitis; this is distinct from meningitis patients (i.e., hematogenous spread), where CSF concentrations of beta-lactam antibiotics are more likely to be increased because inflamed meninges open the tight junctions, which likely enable increased passive drug diffusion into CSF (12). Given ceftolozane-tazobactam distribution into CSF, we found that the pharmacokinetic data were adequately described using a three-compartment model in the study patients (see Fig. S3 in the supplemental material). There was no significant difference in parameter estimates between patients with and without CNS infections, suggesting that the presence of ventriculitis did not have a significant effect on distribution into CSF.

We determined penetration ratios for ceftolozane (0.2 ± 0.2) and tazobactam (0.2 ± 0.26) from unbound plasma and CSF AUC_0–inf_, unlike most previous studies of other beta-lactam antibiotics, where either concentration ratio or total AUC ratio was used. Penetration ratios estimated with *f*AUC for other beta-lactams appear consistent with our findings, for example, 0.26 ± 0.12 for cefotaxime (13). The use of concentration ratio is particularly misleading as the CSF concentration considerably lags behind those in plasma. The use of total AUC ratio is a far better approach; however, important limitations still exist, particularly for those antibiotics with nonnegligible plasma protein binding. That is, given penetration across the BBB mostly occurs by passive diffusion down the concentration gradient (via paracellular pathways for hydrophilic drugs), the free concentration gradient is the driver of penetration, and, therefore, the *f*AUC_0–inf_ ratio is a more appropriate measure of penetration. The use of total AUC ratio is likely to result in lower penetration ratios than that of free AUC, depending on the degree of protein binding.

The presence of CNS infection/inflammation is another important factor that affects the degree of penetration into the CSF. For hydrophilic antibiotics, diffusion across the BBB occurs mainly through the paracellular pathways, which are normally tight in the absence of infection. An animal model study has shown that the host immune inflammatory response may disrupt up to 15 to 17% of the BBB tight junctions in the presence of infection (12), increasing the rate of penetration for water-soluble molecules like ceftolozane and tazobactam. This explains why we observed a statistically significant 10-fold higher *f*ACU_0–inf_ in those patients with presumed/confirmed CNS infections (Fig. 4B). Although we anticipated that a non-CNS infection would replicate the likely penetration for ventriculitis where meninges are not inflamed, we observed higher CSF penetration in patients with CNS infections (all were ventriculitis) compared to non-CNS infections (Fig. 5), although results were statistically significant only at a higher α value of 0.1. Studies comparing penetration ratios for other antibiotics in the presence and absence of infection are lacking. The mean ± SD penetration of 6.85% ± 1.56% observed in this study for ceftolozane in patients without CNS infection is comparable to that reported for piperacillin (5.1% ± 3.5%; mean of 7.28% when corrected for 30% protein binding) in the absence of infection (profound inflammation) (14). We suggest future studies should separately examine the extent of penetration in the presence and absence of inflammation/infection and meningitis compared with ventriculitis.

The observed low penetration ratios translate into suboptimal exposure in the CSF (Tables 3 and 4). Doses higher than 4.5 g q8h appear necessary to achieve the minimum exposure targets of 40% *f*T_>MIC_ for ceftolozane. However, higher exposures of 100% *f*T_>MIC_ may be advantageous given the relative inefficiency of immune response within the CSF (10, 11), suggesting that higher doses of unknown safety are required to maximize microbiological outcomes. Therefore, with currently approved safe doses, monotherapy with ceftolozane-tazobactam may be inadequate for treatment of Gram-negative/ventriculitis unless the etiologic bacterium has a very low MIC (e.g., ≤0.25 mg/liter).

**TABLE 4:** Probability of attaining previously proposed PK/PD targets for tazobactam at steady state^*a*^

Ceftolozane-tazobactam dose (2:1 ratio)	Probability (%) by PK/PD target for:					
	Plasma exposure			CSF exposure		
	20% *f*T_>1 mg/liter_	50% *f*T_>2 mg/liter_	100% *f*T_>4 mg/liter_	20% *f*T_>1 mg/liter_	50% *f*T_>2 mg/liter_	100% *f*T_>4 mg/liter_
3.0 g q8h	100	71	21	12	1	0
3.0 g LD + 9.0 g CI	100	100	86	9	2	0
4.5 g q8h	100	81	26	18	4	0
4.5 g LD + 13.5 g CI	100	100	100	17	3	0

a PK, pharmacokinetic; PD, pharmacodynamic; q8h, every 8 h, intermittent infusion (1 h); % f*T*_>MIC_, percentage of time free drug concentration is greater than the given MIC; LD, loading dose over 1 h; CI, continuous infusion over 24 h.

With currently approved doses, the use of ceftolozane-tazobactam in Gram-negative CNS infection could be considered part of a combination therapy approach with other active antibiotics. In a case report by Frattari et al. (9), a case of XDR P. aeruginosa meningitis was successfully treated with a triple combination of ceftolozane-tazobactam (3.0 g q8h), fosfomycin (4 g q6h), and rifampin (600 mg q6h). The ceftolozane-tazobactam MIC was 3 mg/liter (Etest), for which, according to our dosing simulations (Fig. 6), therapeutic exposure targets are unlikely to be met with ceftolozane-tazobactam monotherapy. The authors did not measure fosfomycin MIC or report the rifampin MIC for the XDR P. aeruginosa. However, it is likely that the synergistic action of the three antibiotics played a role, given previously reported synergy of fosfomycin and rifampin in tandem with other antibiotics (15, 16).

Of note, simulated tazobactam exposures in CSF were extremely low even after a tazobactam dose of 1.5 g q8h (Table 4). Although a clear target for tazobactam exposure is lacking, the probability of achieving a previously recommended target of 20% *f*T_>1 mg/liter_ or 50% *f*T_>2 mg/liter_ was less than 20%. In an *in vitro* study (17) examining tazobactam exposure in combination with ceftolozane, 35, 50, and 70% *f*T_>MIC_ of tazobactam were associated with stasis and 1-log and 2-log CFU reductions. The probability of attaining these targets at the breakpoint (MIC, 4 mg/liter) was nearly zero for the simulated dosing regimens. Similarly, the probability of attaining 100% *f*T_>4 mg/liter_ was zero, which means that the constant concentration of 4 mg/liter used during *in vitro* susceptibility testing is not achieved *in vivo*. In other words, *in vitro* susceptibility results may not relate to *in vivo* susceptibility within the CSF. Therefore, ceftolozane-tazobactam MIC values should be carefully interpreted in the context that *in vitro* susceptibility does not guarantee clinical outcomes in CNS infections in particular.

The interpretation of the current data is also subjected to the limitations of this study. An important limitation is that the pharmacokinetics (PK) in the CSF could be variably affected by the presence of EVD. In shunt CSF, flow rates are variable between patients and within a patient and may not relate to the amount of CSF produced (18). This would limit the generalizability of PK estimates. Another factor affecting generalizability is the small sample size, with only 50% of the population diagnosed with a confirmed/suspected CNS infection, where increased penetration may be likely. The PK/PD analysis was also limited by the lack of data on exposures that define safety thresholds such that increased doses could be recommended to achieve the desired exposure at the predicted low penetration ratios. Finally, we have used first-dose data to develop a PK model and predict steady-state exposure; while this is an acceptable approach, we recommend that future studies confirm steady-state exposures from samples taken at steady state.

In conclusion, the current maximal dose of ceftolozane-tazobactam (3.0 g q8h) does not provide adequate CSF exposure of ceftolozane unless the MIC for the causative pathogen is very low (≤0.25 mg/liter). However, tazobactam exposures remain far lower than the fixed 4-mg/liter concentration used for *in vitro* MIC testing; therefore, the susceptibility of even low-MIC pathogens causing CSF infection remains questionable. Future research should study potential solutions, including whether higher doses can safely be administered via intravenous infusion to achieve adequate CSF exposure, whether alternative routes of administration, such as intrathecal/intraventricular administration, are required, or if synergistic/additive combination with other antibiotics enables the use of standard ceftolozane-tazobactam doses of known safety in the treatment of CNS infections.

## MATERIALS AND METHODS

### Study design, setting, and patients.

This prospective observational pharmacokinetic study was conducted at the University of Queensland Centre for Clinical Research, Brisbane, Australia. Patients were recruited from the quaternary referral intensive care unit (ICU) of the Royal Brisbane and Women’s Hospital (RBWH). The University of Queensland (2017000698) and the RBWH (HREC/17/QRBW/117) human research ethics committees granted ethics approval.

Adult patients (≥18 years) admitted to the ICU of the RBWH were enrolled if they had an indwelling external ventricular drain (EVD) and informed consent was obtained. Patients were excluded if they had a history of allergy to penicillins and/or cephalosporins, they were receiving renal replacement therapy or had estimated creatinine clearance of <10 ml/min, and were receiving piperacillin-tazobactam or had received it in the 7 days before the time of screening.

### Drug administration and sample collection.

All patients received a single dose of 3.0 g ceftolozane-tazobactam (2:1 ratio) administered via intravenous infusion over 1 h. Arterial blood samples (1 to 2 ml) were collected first, prior to dose administration, and serially after the dose at 15 min, 45 min, 75 min (after 15 min of line flushing), 2 h, 3 h, 4 h, 5 h, 6 h, 7 h, 8 h, and 24 h. CSF samples (0.5 to 1 ml) were simultaneously collected from the EVD prior to dose administration at 2 h, 4 h, 6 h, 8 h, and 24 h. The drip chamber of the EVD system was emptied about 30 min before the first CSF sample collection and after each sample collection. In addition, the volume of CSF drainage during the 8 h of sampling was recorded.

### Clinical data.

The Research Electronic Data Capture (REDCap) web application was used to develop an electronic case report form and collect clinical data. These data included patient demographics; physical examination, including vital signs; ICU and hospital admission and discharge dates and times; Acute Physiology and Chronic Health Evaluation II (APACHE II) score; sequential organ failure assessment (SOFA) score at ICU admission; presence of mechanical ventilation; renal function markers (serum creatinine concentration and urinary creatinine clearance); liver laboratory test results (alanine aminotransferase, aspartate aminotransferase, alkaline phosphatase, gamma glutamyl transferase, international normalized ratio, and bilirubin); medication list on days of sampling; antibiotic data, including type, dose, dosing interval, duration of infusion, and other antibiotics administered on the day of sampling; and infection data (organisms isolated and sample type, MIC if available).

### Ceftolozane-tazobactam assay.

An ultrahigh-performance liquid chromatography-tandem mass spectrometry (UHPLC-MS/MS) method was developed on a Shimadzu Nexera2 UHPLC system coupled to a Shimadzu 8050 triple-quadrupole mass spectrometer (Kyoto, Japan) for unbound ceftolozane and tazobactam measurement. Test samples were assayed alongside matrix-matched calibrators and quality controls, and batches were subjected to acceptance criteria (U.S. FDA, 2018). Sample preparation involved isolation of free (non-protein-bound) fractions in plasma and CSF by ultrafiltration at 37°C using Centrifree devices (Merck, Darmstadt, Germany) prior to analysis. Sample (10 μl) was spiked with phosphate-buffered saline (pH 7.4) and internal standard (sulbactam and stable isotope-labeled cefazolin) and treated with acetonitrile. The stationary phase was performed in a C_18_ Ultra IBD, 100- by 2.1-mm, 3-μm column (Restek, Bellefonte, PA) operated at room temperature. Mobile phase A was 0.1% (vol/vol) formic acid in 10 mM ammonium formate, and mobile phase B was 100% acetonitrile with 0.1% (vol/vol) formic acid. The mobile phase was delivered with a gradient from 15% to 50% B at a flow rate of 0.3 ml/min for a 5-min run time and produced a backpressure of approximately 2,800 lb/in^2^. Ceftolozane was monitored by positive mode electrospray at multiple reaction monitoring (MRM) of 667.00→199.15. Labeled cefazolin was monitored in positive mode at 457.85→326.05. Tazobactam and sulbactam were monitored by negative-mode electrospray at MRMs of 299.20→138.00 and 232.20→140.00, respectively. The method met U.S. FDA validation criteria; between-run and within-run precision and accuracy were within 10%.

### Population pharmacokinetic model building.

Pmetrics version 1.5.2, a package for R, was used for pharmacometric analysis based on the nonparametric adaptive grid algorithm (NPAG). Two- and three-compartment structural models were initially tested with linear elimination from the central compartments and linear and/or Michaelis-Menten distribution from the central to a peripheral/CSF compartment(s). An additive or multiplicative error model available within Pmetrics was tested with each of the structural models. The error models are based on the standard deviation (SD) of observations that take the form error = (SD^2^ + λ^2^)^0.5^ and error = SD × γ, where λ and γ are process noises associated with the observations. In addition, error associated with assay was modeled as a second-degree polynomial function of the SD of measurements.

Biologically plausible covariates were screened using built-in regression analysis in Pmetrics and observation of covariate versus model parameter plots. Available covariates selected for screening included age, sex, weight, height, albumin concentration, serum creatinine, urinary creatinine clearance, volume of CSF collected from the ventricular drain, APACHE II score, SOFA score, and the presence of CNS infection. Potential covariates were tested on primary model parameters with either linear or exponential models.

### Model diagnosis.

Diagnostic plots included observed versus population or individual predicted concentrations, visual predictive check plots, and normalized prediction distribution errors (NPDE) versus time or observation plots. Statistical comparison of modes was based on the regression coefficient of observed versus predicted concentrations, bias [defined as the mean weighted error of predicted minus observed concentrations, i.e., Σ(predicted − observed/SD)/*N*], imprecision (defined as the bias-adjusted, mean weighted squared error of predicted minus observed concentration, i.e., Σ [(predicted − observed)^2^/(SD)^2^]/*N* − Σ(predicted-observed)/SD/*N*, where *N* is the number of observations/predictions), and objective functions, including the log likelihood ratio (LLR) test for the nested models, Akaike information criterion (AIC), and Bayesian information criterion (BIC). The LLR chi-square test was used for statistical comparison of nested models (a *P* value of <0.5 was considered significant).

### Dosing simulations.

Monte Carlo dosing simulation (*n* = 1,000) were performed in R. The simulator in Pmetrics draws random samples repeatedly from a joint parameter probability distribution from NPAG. From each sampled set of parameter values, observations are calculated based on a predefined template of the input, covariates, and output measurement times. Simulations were performed for ceftolozane-tazobactam (2:1 ratio) regimens of 3.0-g intermittent infusion (over 1 h) q8h, 4.5 g q8h, 3.0-g loading dose over 1 h followed by 9.0-g continuous infusion over 24 h, and 4.5-g loading dose followed by 13.5-g continuous infusion over 24 h. Dosing simulations were performed up to steady state from 0 to 144 h.

The probability of target attainment (PTA) was estimated primarily for ceftolozane PK/pharmacodynamic (PD) exposure targets of 40% *f*T_>MIC_ (19, 20) and for 60% and 100% *f*T_>MIC_. For tazobactam exposures, PTA was estimated for suggested targets of 20% *f*T_>1 mg/liter_ (20% of the time above the minimum effective concentration of 1 mg/liter) (21) and 50% *f*T_>2 mg/liter_ (22) and 100% *f*T_>4 mg/liter_ to assess exposure relative to the *in vitro* susceptibility testing condition of fixed 4-mg/liter tazobactam concentration (23). PTA was estimated based on 24-h exposure at steady state from 120 to 144 h.

Cumulative fractional response (CFR) was estimated for ceftolozane, using P. aeruginosa EUCAST MIC distributions (accessed 27 May 2020) within the susceptible category (≤4 mg/liter). A CFR value of ≥85% was considered acceptable. The following equation was used for CFR calculation.

(1) $$\text{CFR}=\overset{4}{\underset{i=0.125}{\sum }}{\text{PTA}}_{i}\times F_{i}$$

where *i* is MIC category ranging from 0.125 to 4 mg/liter, PTA*_i_* is the PTA at each MIC category, and *F_i_* is the fraction of the bacterial population at each MIC category.

### Additional statistical evaluation.

In addition to inbuilt tools within Pmetrics for R, GraphPad Prism (version 8.3.1; GraphPad Software, LLC) was used to generate plots from simulation outputs and perform Welch’s *t* test comparison of CSF penetration ratio between patients with CNS infection versus those without.

## Supplementary Material

Supplemental file 1
