# Supplementary material for: Cerebrospinal Fluid Penetration of Ceftolozane-Tazobactam in Critically Ill Patients with an Indwelling External Ventricular Drain
Source: Antimicrob Agents Chemother. 2020 Dec 16;65(1):e01698-20. doi: 10.1128/AAC.01698-20 (PMC7927828; doi:10.1128/AAC.01698-20)
Supplement: Supplemental file 1 [file AAC.01698-20-s0001.pdf]

Supplementary File

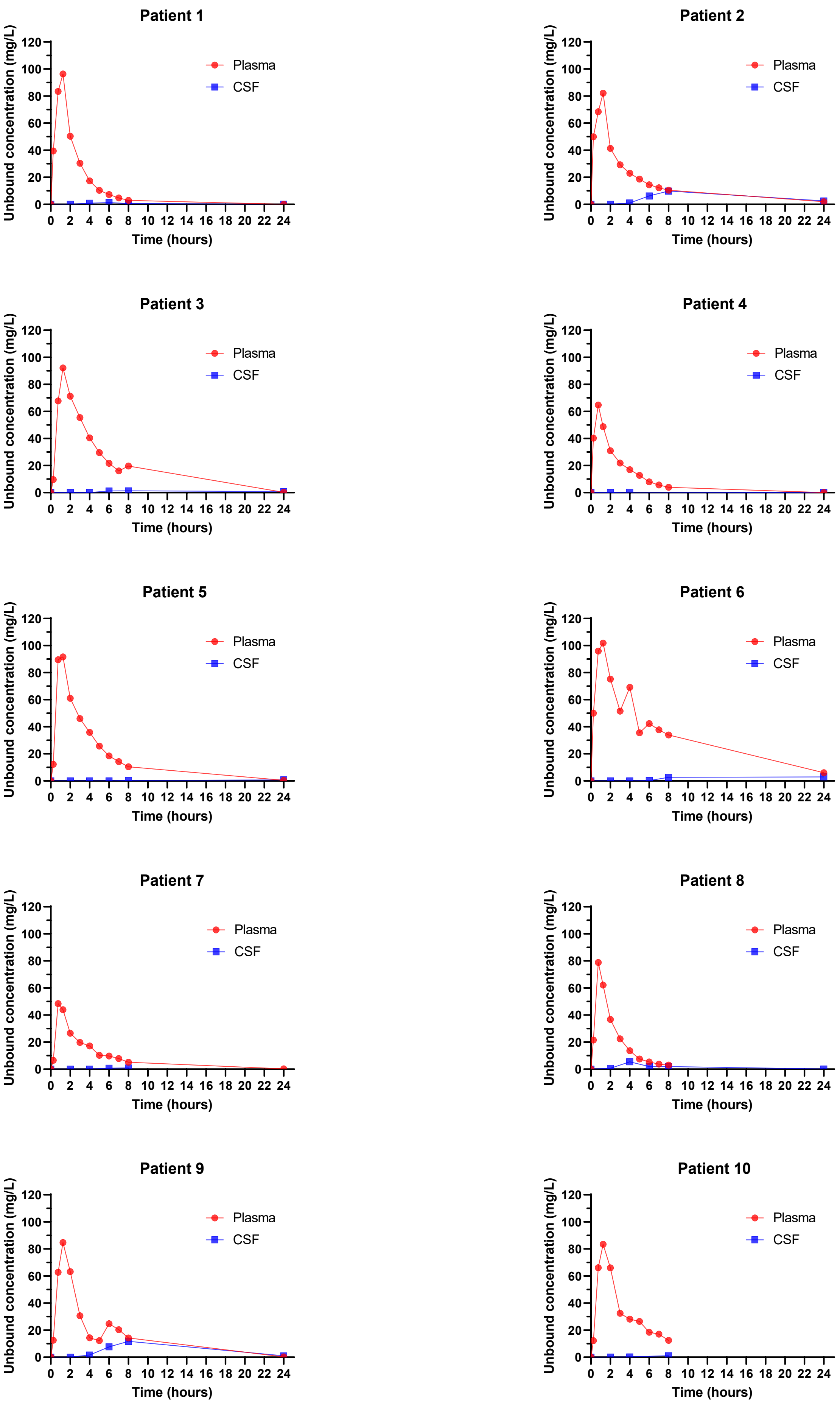

Figure S1. Plasma versus cerebrospinal fluid ceftolozane concentration-time profiles observed in each patient.

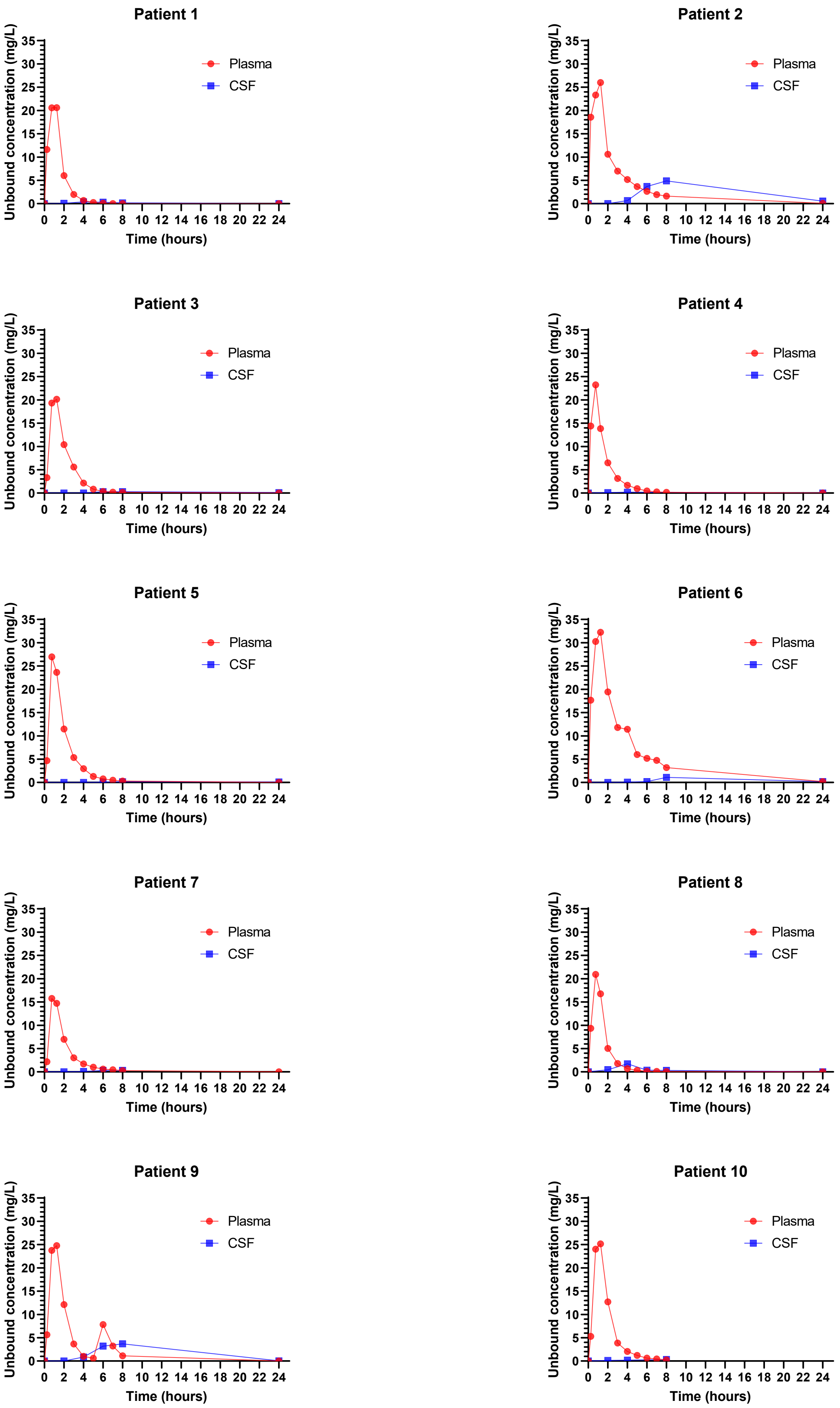

Figure S2. Plasma versus cerebrospinal fluid tazobactam concentration-time profiles observed in each patient.

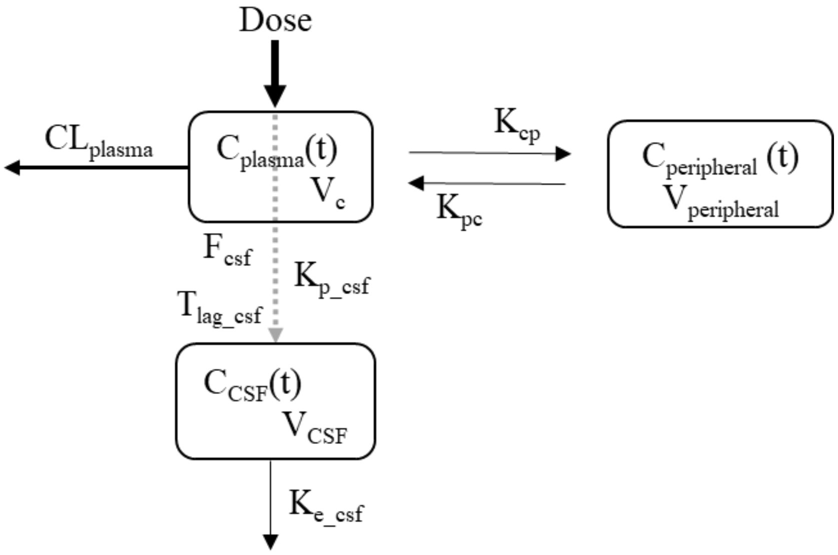

Figure S3. Schematic representation of the final structural model. $C_{plasma}(t)$, plasma concentration at time t; Vc, apparent central volume of distribution; CL_{plasma}, total clearance from plasma; K_{cp}, rate constant for the transfer from the central compartment to the peripheral compartment; K_{pc}, rate constant for the transfer from the peripheral compartment to the central compartment; $C_{peripheral}(t)$, concentration in the peripheral compartment at time t; $V_{peripheral}$, volume of distribution of the peripheral compartment; K_{p_csf}, rate constant for penetration into the CSF compartment; $C_{CSF}(t)$, CSF concentration at time t; V_{CSF}, apparent volume of distribution of the CSF compartment; K_{e_csf}, rate constant for elimination from the cerebrospinal fluid (CSF) compartment; T_{lag_csf}, lag time for penetration into the CSF compartment; F_{csf}, bioavailability in the CSF compartment.

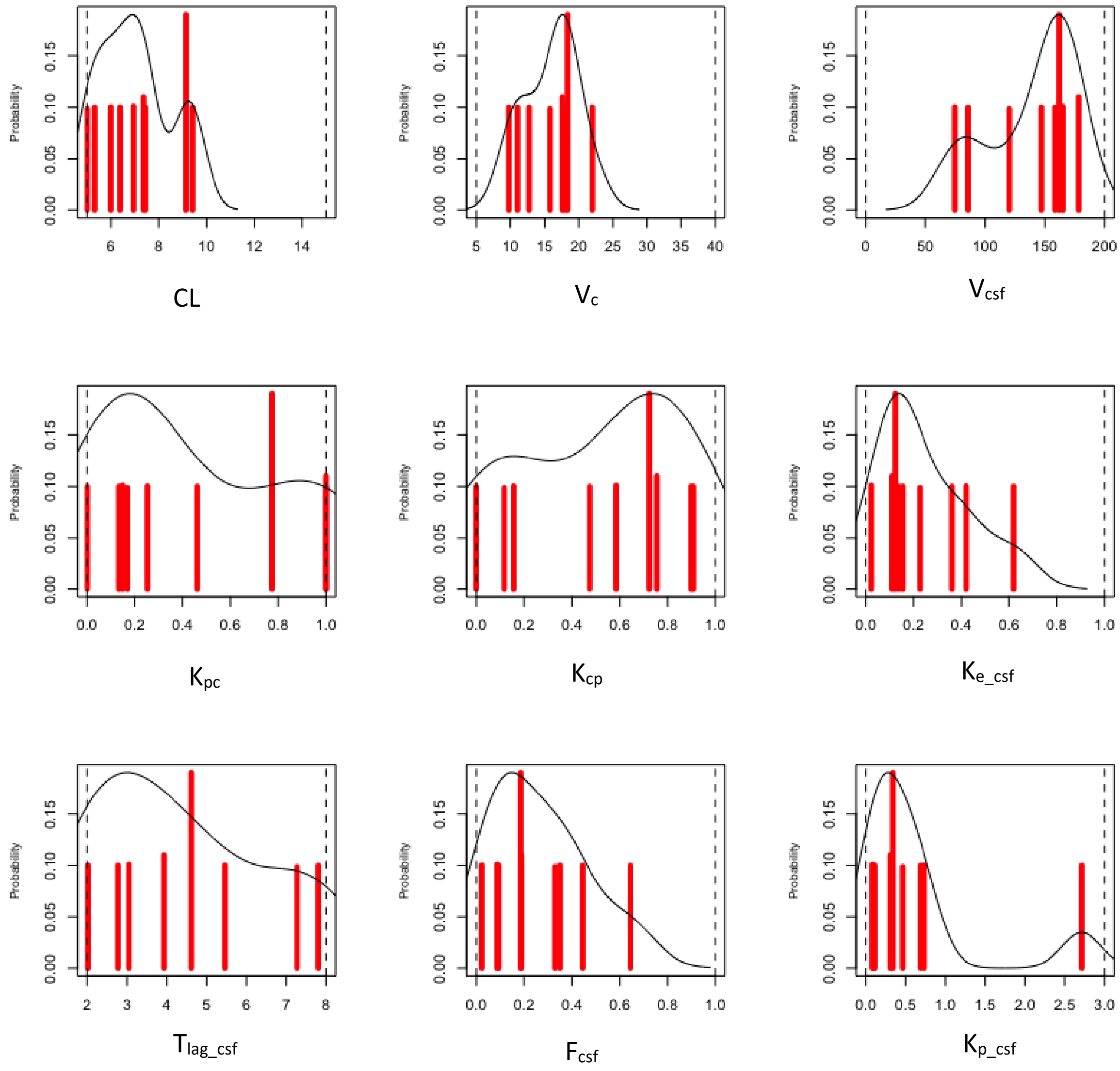

Figure S4. Parameter distribution plot for ceftolozane. The solid black curve gives the kernel density estimate

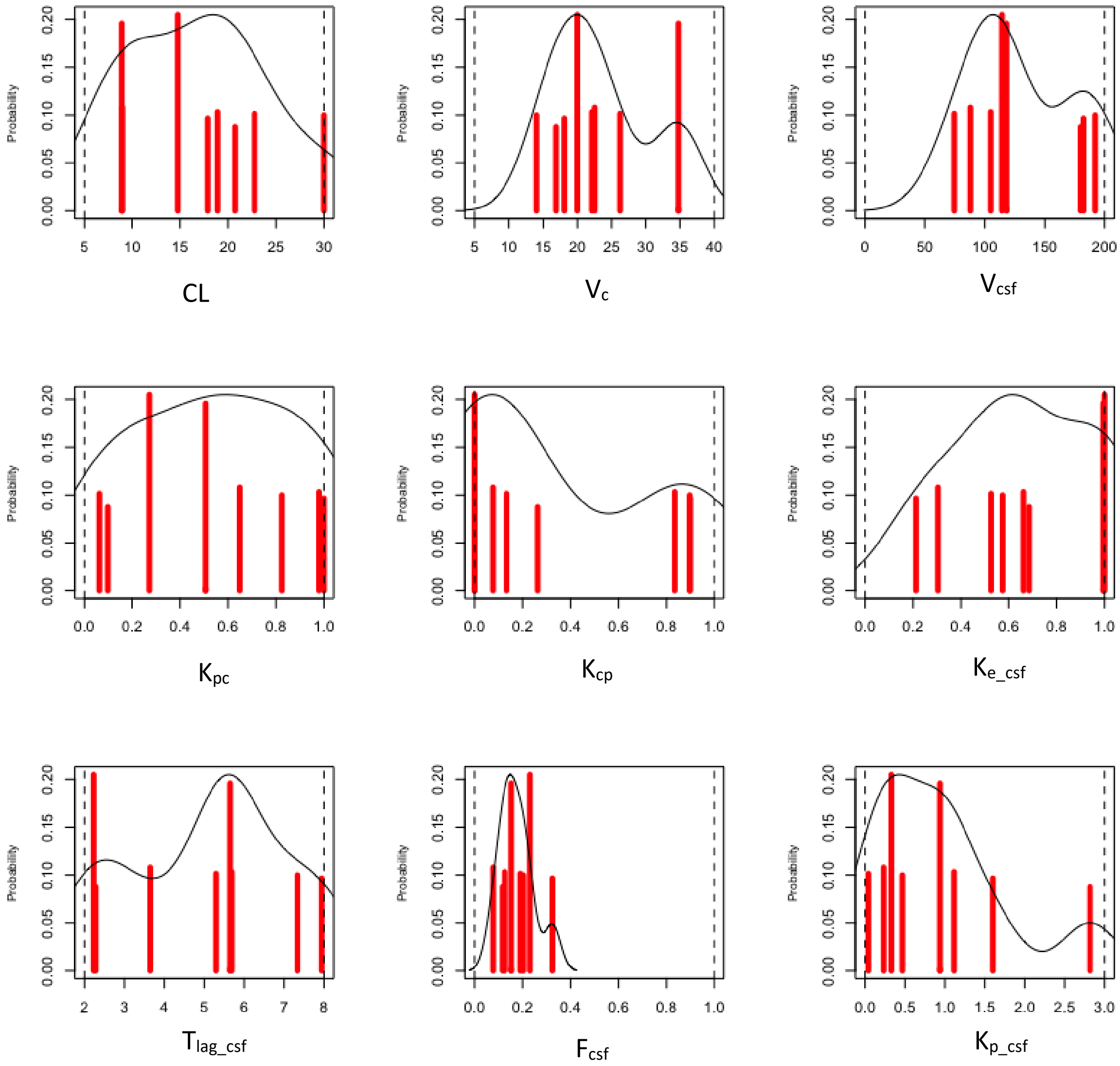

Figure S5. Parameter distribution plots for tazobactam. The solid black curve give the kernel density estimate.
